# Clustering of serum biomarkers involved in post-aneurysmal subarachnoid hemorrhage (aSAH) complications

**DOI:** 10.1007/s10143-023-01967-9

**Published:** 2023-03-03

**Authors:** Igor Fischer, Shafqat Rasul Chaudhry, Daniel Hänggi, Sajjad Muhammad

**Affiliations:** 1 Department of Neurosurgery, Medical Faculty and University Hospital Düsseldorf, Heinrich-Heine-University Düsseldorf, Moorenstraße 5, 40225 Düsseldorf, Germany; 2 Department of Pharmacy, Obaid Noor Institute of Medical Sciences (ONIMS), Mianwali, 42200 Punjab Pakistan

**Keywords:** Stroke, SAH, CVS, Neuroinflammation, Serum biomarkers, Clustering

## Abstract

Post-aSAH sequela is characterized by the onset of life-threatening complications along with the upregulated underlying inflammation. Cerebral vasospasm (CVS) is one of the most frequent complication after aSAH, which contributes majorly to delayed cerebral ischemia and poor clinical outcome. The objective of this study was to identify the clusters of serum biomarkers that are associated with cerebral vasospasm (CVS) after suffering from aneurysmal subarachnoid hemorrhage (aSAH). In this single-center study, serum concentrations of 10 potential biomarkers, together with clinical and demographic parameters, for 66 aSAH patients were recorded within 24 h after aSAH. The dataset was split into a training set (43 patients) and a validation set. Correlation heatmaps for both datasets were computed. Variables with inconsistent correlations on the two subsets were excluded. Clusters of relevant biomarkers were identified on the complete set, separately for patients who developed post-aSAH CVS and those who did not. Two clusters were found to be specific for patients who suffered from CVS: mitochondrial gene fragments (cytochrome B (Cyt-B), cytochrome C oxidase subunit-1 (Cox-1), displacement loop (D-loop), and IL-23, and the other one, containing IL-6, IL-10, age, and Hunt and Hess score. Clusters of serum biomarkers, analyzed within 24 h of the onset of aSAH, days before the CVS development, are expressed differently in patients suffering from post-aSAH CVS, compared to patients without CVS. This suggests that these biomarkers may be involved in the pathophysiological processes leading to CVS and may be used as its early predictors. These interesting findings are potentially highly relevant for the management of CVS and call for validation on a larger sample of patients.

**Supplementary Information:**

The online version contains supplementary material available at 10.1007/s10143-023-01967-9.

## Introduction

Subarachnoid hemorrhage (SAH) is a debilitating and often fatal disease, with an estimated worldwide incidence between five and 10 per 100,000 person-years, with high regional variability [1, 2]. The most common cause of non-traumatic subarachnoid hemorrhage is the rupture of an intracranial aneurysm in about 75–85% of the cases [3, 4]. The mortality is high, reportedly reaching 42% [5] or even 50% [6], and many survivors suffer from permanent disability, with about 50% dependency rate [7].

The mortality and the morbidity result not only from the initial hemorrhage, but also from post-aSAH complications, which are frequent [8, 9]. aSAH is known to induce a peripheral immune response, resulting in accumulation of immune cells in brain parenchyma [10] and release of cytokines [11]. The resulting inflammation has been shown to correlate with poor clinical status at admission [12]. Successful management of post-aSAH inflammation may therefore offer therapeutic benefits [13].

Cerebral vasospasm (CVS) is the most feared and frequent secondary neurologic complication, with an overall incidence of 50 to 90% and accounting for between 10 and 23% of the deaths [14, 15]. The mechanistic underpinnings of CVS are still the subject of intensive research. Although it is established that the CVS is not the sole player that leads to poor clinical outcome after aSAH, it is a major contributor to delayed cerebral ischemia (DCI) which is known to cause poor clinical outcome after aSAH [16, 17]. The large number of potential CVS contributing factors to be investigated pose several serious practical challenges. This problem is not specific to CVS. Pathophysiological processes are complex and convoluted, and often depend on many physiological and metabolic variables that are subject to vary depending upon the complex interplay of environmental and heterogeneous genetic backgrounds of the patients. On one hand, the modern diagnostic monitoring and laboratory technology offers the possibility to collect large amounts of such data and this has become a common practice in medical research. On the other hand, it is very time-consuming to recruit a large group of patients or collect a large number of independent probes. Consequently, medical datasets are often very “wide,” having few observations, but many variables per observation. This is problematic because each new variable introduces an additional parameter which should be fitted to into the data.

From the data-scientific point of view, analysis of sparse, high-dimensional data faces severe difficulties. Parameters pertaining to a single patient are likely to be dependent, but, even if they were not, a large parameter space with a small number of observations inevitably increases the probability of encountering spurious associations or correlations or, in other words, stumbling over false positives. Therefore, every serious analysis of such data must include some kind of dimensionality reduction. In some cases, where established theories or prior research provide enough information, human experts may exploit their domain knowledge and manually eliminate irrelevant variables. Sophisticated, domain-agnostic methods from machine learning also exist, but require large amounts of data.

In our study, we applied a clustering approach over correlation coefficients of the collected clinical measures and blood serum biomarkers to identify those involved in CVS. We used two separate datasets to validate the results and minimize the danger of false positives.

## Methods

### Ethics statement

The study was performed according to the guidelines of the Helsinki declaration and was approved by the local ethics committee of the medical faculty of the University of Bonn, Germany (reference number: LfD 138/2011). The informed consent was obtained by the treating neurosurgeon. The manuscript was prepared according to the STROBE guidelines.

### Study type and population

The study was performed retrospectively and the data were collected in two phases: first, in 2012–2014, in which 52 patients were included, and the second, with 28 patients, in 2015–2016. The data from the first phase were used as the training set, and the latter for validation. The patients presenting within 24 h of aSAH were only considered for sampling. The patients with age ≤ 18 years, ischemic stroke, traumatic brain injury, onset of symptoms beyond 24 h, SAH due to arteriovenous malformations or vasculitis, pregnancy, signs of eminent death, or those who did not provide consent were excluded from the study. After excluding the patients with missing data, 43 patients remained in the training set and 23 in the validation set. The demographic variables and their corresponding descriptive statistics are listed in Table 1. No significant differences between the demographic parameters of the training and the validation sets were observed.

**Table 1:** Demographics, clinical and outcome scores

	Statistic	Total	Training	Validation	Stat. test	*p*-value
Set size	*N* (%)	66 (100%)	43 (65%)	23 (35%)	-	-
Sex = 1	*N* (%)	25 (38%)	14 (33%)	11 (48%)	Chi-squared	0.34
Age (years)	mean (sd)	56.7 (12.2)	58.5 (12.3)	53.3 (11.4)	*t*-test	0.097
Fisher	*N* (%)	= 1: 1 (2%) = 2: 2 (3%) = 3: 54 (82%) = 4: 9 (14%)	= 1: 1 (2%) = 2: 2 (5%) = 3: 34 (79%) = 4: 6 (14%)	= 1: 0 = 2: 0 = 3: 20 (89%) = 4: 3 (13%)	Chi-squared	0.63
Hunt and Hess	*N* (%)	= 1: 5 (8%) = 2: 23 (35%) = 3: 18 (27%) = 4: 12 (18%) = 5: 8 (12%)	= 1: 1 (2%) = 2: 18 (42%) = 3: 13 (30%) = 4: 7 (16%) = 5: 4 (9%)	= 1: 4 (17%) = 2: 5 (22%) = 3: 5 (22%) = 4: 5 (22%) = 5: 4 (17%)	Chi-squared	0.1
GOS	*N* (%)	= 1: 4 (6%) = 2: 8 (12%) = 3: 18 (27%) = 4: 4 (6%) = 5: 32 (48%)	= 1: 2 (5%) = 2: 5 (12%) = 3: 13 (30%) = 4: 3 (7%) = 5: 20 (46%)	= 1: 2 (9%) = 2: 3 (13%) = 3: 5 (21%) = 4: 1 (4%) = 5: 12 (52%)	Chi-squared	0.89
mRS	*N* (%)	= 0: 2 (3%) = 1: 24 (36%) = 2: 7 (11%) = 3: 6 (9%) = 4: 12 (18%) = 5: 11 (17%) = 6: 4 (6%)	= 0: 1 (2%) = 1: 16 (37%) = 2: 3 (7%) = 3: 6 (14%) = 4: 7 (16%) = 5: 8 (19%) = 6: 2 (5%)	= 0: 1 (4%) = 1: 8 (35%) = 2: 4 (17%) = 3: 0 = 4: 5 (22%) = 5: 3 (13%) = 6: 2 (9%)	Chi-squared	0.43
CVS	*N* (%)	36 (55%)	23 (53%)	13 (57%)	Chi-squared	1

### Variable definition and data collection

SAH was determined by computerized tomography (CT) scan. Patients were clinically assessed and/or, if possible, daily screened by transcranial Doppler ultrasound (TCD) for vasospasm. On suspicion, CT angiography (CT-A) and CT perfusion (CT-P) were performed to confirm CVS. Vasospasm was defined as a mean transit time (MTT) of over 5 s in CT-P or as reduced vessel diameter by more than 50% in digital subtraction angiography (DSA). MTT was computed from the CT-P as the ratio of cerebral blood volume (CBV) and cerebral blood flow (CBF), which, in turn, were computed by deconvoluting the tracer response signal from the arterial input function (AIF). The deconvolution was performed using singular value decomposition (SVD) (STROKETOOL-CT, Digital Image Solutions, Frechen, Germany). The decision whether to rely on DSA or CT-P was based on clinical considerations. DSA allows for direct confirmation of CVS, while in CT-P only its effects are measurable. On the other hand, CT-P visualizes the brain cross-section, while DSA only provides a flattened projection. Patient outcome at discharge was quantified using the Glasgow outcome scale (GOS) and modified Rankin scale (mRS).

Besides the demographic and the clinical data, serum concentrations of 10 potential biomarkers were quantified as described elsewhere [18–23]. These included damage-associated molecular patterns (DAMPs) (HMGB1, mitochondrial DNA gene fragments such as cytochrome B (Cyt-B), D-loop, and cytochrome c oxidase subunit-1 (Cox-1)), pro- and anti-inflammatory cytokines (IL-6, IL-17, IL-23, IL-10, CCL5), and leukocytes. Blood samples collected within 24 h after aSAH and processed using a previously described protocol [23] were used for this study. Also, the information whether and on which day after aSAH the patient developed CVS was recorded.

### Data analysis

The relationships between demographic variables on one hand and the biomarkers on the other hand were tested using suitable statistical methods: linear regression for biomarkers vs. age, ANOVA for biomarkers vs. ordinal variables (Fisher score, Hunt and Hess score, GOS score, and mRS) and *t*-test for biomarkers vs. binary variables (sex and whether the patients suffered CVS). Altogether, 70 tests were performed. In seven cases, the *p*-value was below 0.05: Cox-1 vs. Fisher score, IL-10 vs. Fisher, Hunt and Hess, GOS, and mRS scores, Cyt-B vs. sex, and HMGB-1 vs. CVS. One should bear in mind, however, that in multiple testing the danger of false positives rises greatly. When applying Bonferroni correction, none of the relationships was significant.

To ensure comparability of the training and the validation sets, the variables pertaining to demographics and clinical status/outcome were compared using *t*-test for the continuous variable (age) and chi-squared test for the remaining nominal and ordinal variables.

The ten biomarkers and the patient age were transformed using the Yeo-Johnson power transform [24] and normalized to zero mean and unit standard deviation in order to obtain an approximately standard normal distribution. Besides the transformed biomarkers and the age, the Hunt and Hess score was used for analysis. Fisher score was excluded, as it was determined to be highly redundant with the Hunt and Hess score, and the outcome scores were discarded as considered irrelevant as the predictors of CVS during hospital stay.

To identify variables—biomarkers, demographic, and clinical parameters—relevant in the development of the CVS, we computed correlation heat maps for all 12 × 11 / 2 = 66 pairs of variables, separately on the training and the validation sets. We included the Hunt and Hess score in the analysis due to its clinical importance, despite it being an ordinal variable. Still, correlations involving it should be considered with caution. To minimize the danger of encountering false positives, we excluded from further analysis any variables, which showed inconsistent correlations on the two subsets. For the purpose of the study, “inconsistent” correlation was defined as having the opposite sign or differing by more than 0.25 on the two sets. Clusters of relevant biomarkers were identified on the complete (training + validation) set as having correlations of at least ± 0.25 and sharing at least three predictors. Clustering was performed separately for patients who developed post-aSAH CVS and those who did not. For visualization, we also mapped the variables onto a 2D coordinate system using a non-linear dimensionality reduction, which attempts to place correlated variables geometrically close to each other.

All computations were performed in Python (www.python.org), using NumPy [25], SciPy [26], seaborn [27], and Scikit-learn [28] libraries.

## Results

A summary of the demographic data is given in Table 1 and a summary of the serum biomarker values in Table 2.

**Table 2:** Serum biomarkers summary for the cohort

Biomarker	Range: mean (sd)
HMGB1	14.2 (21.6)
Cyt-B	12.8 (21.8)
D-Loop	10.5 (22.5)
Cox-1	10.4 (22.6)
IL-6	11.7 (22.1)
IL-17	12.8 (22)
IL-23	44.5 (51.2)
IL-10	95.3 (168)
CCL5	17,300 (18,600)
Leukocytes	19 (20.5)

### Correlations on the training and the validation sets

Some variables, such as Cyt-B and Cox-1, were consistently highly correlated in both datasets, i.e., training and validation sets. Others, such as IL-17 and HMGB1, appeared moderately positively correlated on the training set, but strongly negatively correlated on the validation set. Consistently high correlations of the same sign in the both sets appeared rarely. Figure 2 highlights the correlations, which were consistently high, in absolute values, in both the training and the validation sets, for the patients who suffered CVS. Figure 4 shows the same for the other group of patients, who were spared from CVS.

### Stable and relevant correlations

**Fig. 1:**
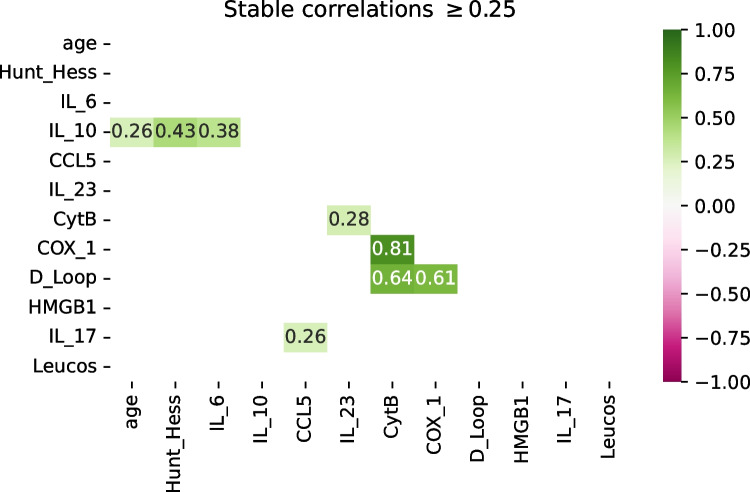
Correlation heatmap of variables which were consistently similar and relevant (i.e., had the same sign and differed by < 0.25 between the training and the validation sets—66 patients altogether—and for which the magnitude of the correlation was ≥ 0.25). Only eight pairs of variables satisfy these conditions. D-loop, cytochrome B (Cyt-B), and cytochrome C oxidase subunit-1 (Cox-1) form a cluster with moderate-to-high correlations, with Interleukin-23 (IL-23) weakly correlated to it. IL-10 shows weak-to-moderate correlation with age, Hunt and Hess score, and IL-6

After removing the pairs with inconsistent correlations in the training and the validation sets, 29 pairs of variables remained. However, most of these correlations were too low to indicate a practically relevant association between the predictors. Removing the pairs of variables with the magnitude of the correlation below 0.25, only 8 pairs of variables were left (Fig. 1). Of these, D-loop, Cox-1, and cytochrome B formed a cluster, with pair-wise correlations > + 0.6 (+ 0.81 for Cyt-B and Cox-1). IL-23 seemed to be loosely associated with this cluster, through its correlation with Cyt-B (+ 0.28). IL-6 and IL-10 were moderately correlated (+ 0.38), and IL-10 also showed some consistent correlation with the Hunt and Hess score (+ 0.44) and, weaker, with age (+ 0.26), respectively. There was a weak correlation between IL-17 and CCL5 (+ 0.26).

### Correlations by CVS status

**Fig. 2:**
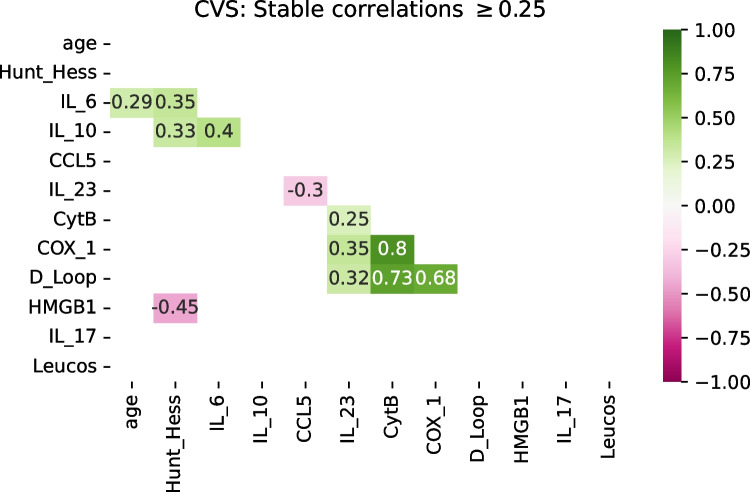
Correlation heatmap of consistently similar and relevant variables for patients who developed CVS (36 patients). The clusters consisting of IL-23, Cyt-B, Cox-1, and D-loop appear approximately in the middle. Near the top left is the cluster consisting of IL-6, IL-10, age, and Hunt and Hess score

**Fig. 3:**
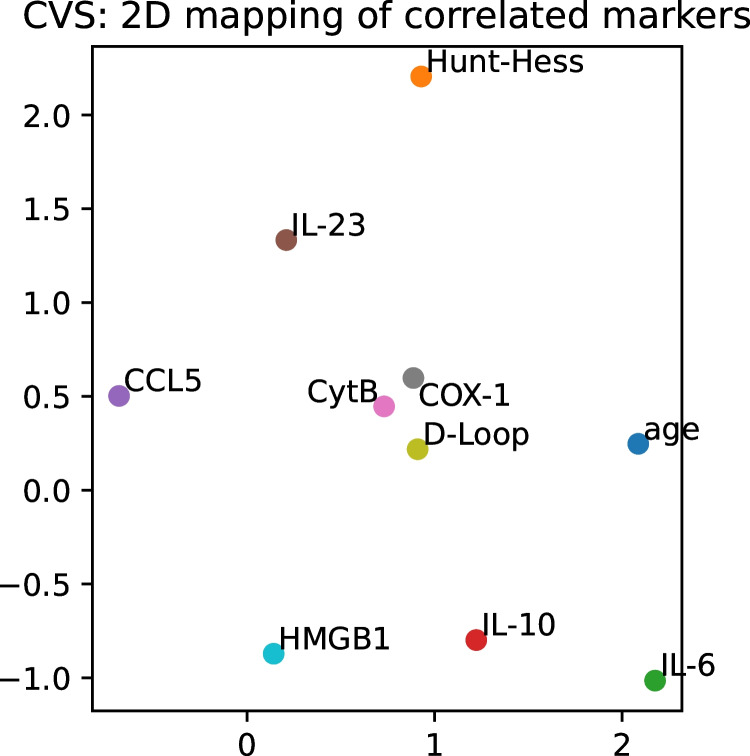
A 2D-mapping of the predictor variables for patients who developed CVS (36 patients). Highly correlated variables tend to appear close to each other on the map, such as Cox-1, Cyt-B, D-loop, and, somewhat further, IL-23. Note, however, that dimensionality reduction inevitably introduces information loss. Hunt and Hess score appears further from age, IL-10, and IL-6 than the correlations between these variables would suggest

The stability of correlations was different between the two groups of the patients, those who developed CVS, and those who did not. Interestingly, for patients who developed CVS, two clusters of variables were identified. One consisted solely of the above mentioned serum markers comprising Cyt-B, Cox-1, D-loop, and weakly associated IL-23, with the correlations ranging from 0.25 to 0.35 between IL-23 and the remaining variables, and 0.68 to 0.80 among the mitochondrial DNA gene fragments. The other cluster comprised IL-6, IL-10, age, and Hunt and Hess scores, with only fair correlations between the variables (0.29–0.40) (Fig. 2). With a loosened criterion for stable correlations, allowing for a difference of 0.3 between the training and the validation sets (instead of 0.25), leukocytes and CCL5 also join this cluster, being weakly negatively correlated with age (*r* = –0.29 and *r* = –0.3, respectively; Supplementary Fig. S4). A 2D mapping of the predictor variables for the patients suffering from CVS is shown in Fig. 3.

**Fig. 4:**
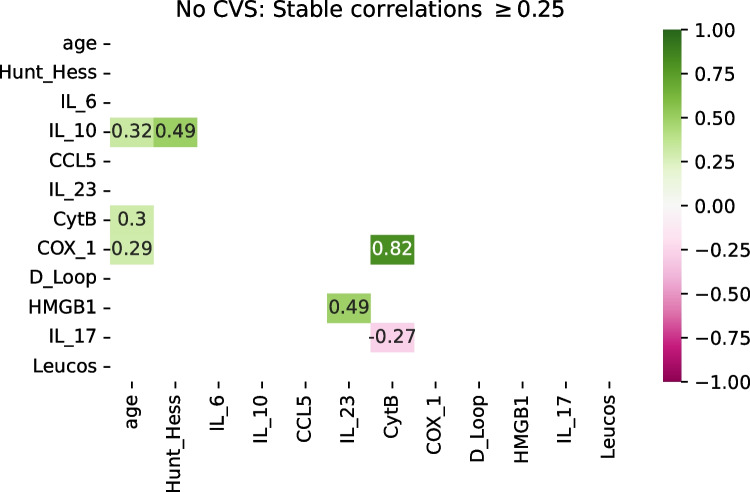
Correlation heatmap of consistently similar and relevant variables for patients who were spared from CVS (30 patients). There are no visible clusters of variables

**Fig. 5:**
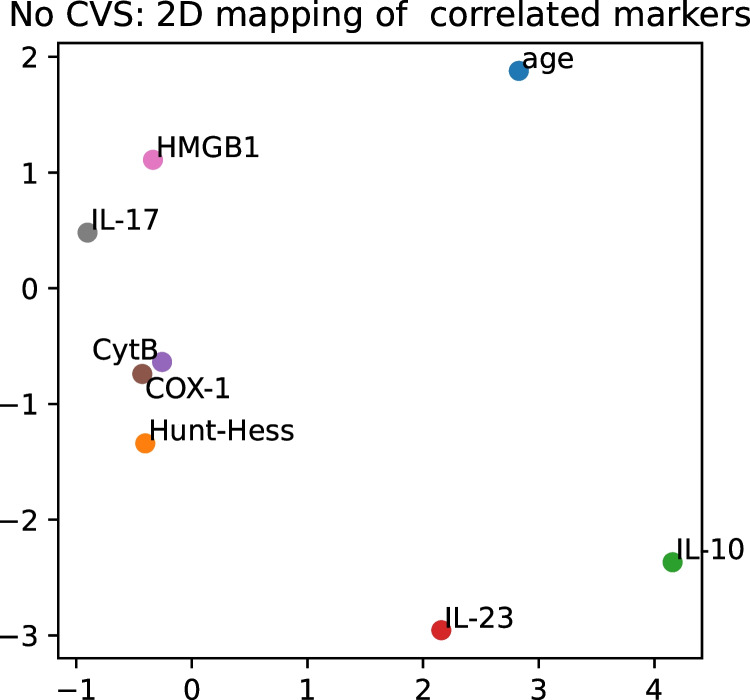
A 2D-mapping of the predictor variables for the patients without CVS (30 patients). Only Cox-1 and Cyt-B are highly correlated and mapped close to each other, and no further clusters are visible

Among the patients who did not develop CVS, no such clusters were observed. Only Cyt-B and Cox-1 showed a very strong correlation (*r* = 0.82), but that could be expected, as both markers represent mtDNA genes. IL-23 was moderately (*r* = 0.49) associated with HMGB1, and IL-10 with Hunt and Hess score. The remaining correlations were weaker (r ≈ ± 0.3) and likely only spurious (Figs. 4 and 5).

Also, in the CVS group, we found a moderate negative correlation between HMGB1 and the Hunt and Hess score (*r* = –0.45) (but see the caveat regarding ordinal variables above). This correlation was absent in the non-CVS group. A further moderate correlation (*r* = 0.4), between IL-6 and IL-10, was observed in the CVS group, but not among the non-CVS patients. The results only slightly changed when considering complete data, with the training and the validation sets combined (Supplementary, Fig. S5).

Linear regression between markers within clusters revealed significantly different relationships between D-loop and Cyt-B for patients who suffered from CVS and those who did not. In both groups, D-loop and Cyt-B were positively correlated. But, for patients who suffered from CVS, a unit increase of Cyt-B coincided with a 61% larger increase in D-loop than in the non-CVS group (*β*_CVS_ = 0.76 vs. *β*_no_CVS_ = 0.47, *p* = 0.02). The relationships between D-Loop and Cox-1 differed slightly above the significance level: A unit increase in Cox-1 was associated with a 55% larger increase of D-loop in patients who suffered from CVS, compared to those who did not (*β*_CVS_ = 0.71 vs. *β*_no_CVS_ = 0.46, *p* = 0.06).

## Discussion

We performed cluster analysis of the clinical parameters and blood serum markers of patients who suffered from aSAH and investigated the differences in clustering for patients who developed CVS and those who did not. General analysis of correlations among the measured serum biomarkers and clinical parameters of aSAH patients showed some interesting correlations. For instance, IL-6 and IL-10 were moderately correlated, which is in line with the previous findings suggesting that IL-10, a master anti-inflammatory cytokine, is always increased in parallel in association with pro-inflammatory cytokines, e.g., IL-6 [29]. Furthermore, IL-6 is known to induce the expression of IL-10 through regulatory mechanisms [30]. IL-10 also showed correlations with Hunt and Hess score and age of the aSAH patients, which corroborates to the previous investigations [18, 29]. A weak correlation was revealed during analysis among CCL5 and IL-17, both of which have been known to increase after aSAH, but their mechanistic links are not exactly known [12, 13, 19, 21].

Intriguingly, we showed that among patients who developed CVS during their hospital stay, some biomarkers, evaluated on day 1, i.e., prior to CVS, were correlated, but remained uncorrelated for patients who did not develop CVS. Therefore, observing simultaneous alterations in the serum levels of these markers might offer an early warning of an imminent CVS.

Analysis of the data with many variables always bears the danger of misinterpreting spurious findings as true relationships, or, in more technical terms, confusing noise for signal. If we consider each variable to be an axis in a coordinate space, the space under consideration can be said to be very high-dimensional, but sparsely populated, due to the low number of observations. This leads to the “curse of dimensionality” [31, 32], which is a widely encountered problem in medical research [33–36].

In our study, we relied on the procedure, which is commonly employed in the statistics and machine learning: splitting the data into a training set and a validation set, and ensuring that the performance is similar on both.

A high correlation between Cox-1 and Cyt-B, which was stable in both groups, is not surprising. Both are the parts of the mitochondrial respiratory chain and play an important role in electron transport [37]. D (displacement)-loop, on the other hand, is a non-coding region and acts as a promoter for both mtDNA strands [38]. The mechanisms which cause its high correlation with the former two markers in the CVS group are still a subject of research. As D-loop and Cyt-B occupy adjacent regions on the mtDNA [39], they are likely to stay together in case of a mitochondrial demise and consequent disintegration of the DNA, which may be associated with CVS. Mitochondrial DNA is a DAMP that upregulates the innate immune responses through TLR-9 activation during sterile systemic inflammatory response syndrome and leads to the release of proinflammatory cytokines [40]. It has already been shown that mtDNA and IL-23 levels are upregulated after aSAH [20, 21]. This is in line with the already established role of IL-23 as a key mediator of inflammation and its role in the secondary brain injury after intracerebral hemorrhage (ICH) [41] and in aSAH [42]; however, its relation to CVS remains to be explored in detail.

## Limitations

In our study, we set a threshold of 0.25 for both the correlation magnitude and the correlation consistency. The value was chosen as a compromise between being too stringent on the correlation magnitude and too lenient on the consistency. On one hand, a higher value, e.g., 0.3, would have filtered out correlations with a lower magnitude, but, on the other hand, it would have allowed for higher discrepancies between the training and the validation sets. While our threshold may seem somewhat arbitrary, simulations show its plausibility. We performed 10,000 simulations, with a random split between the training and validation sets in each one, in a 2:1 ratio. The correlations on the training set, which had the sign consistent with the validation set, were always above 0.25 (Supplementary, Fig. S1). On the other hand, differences in correlations between the training and the validation sets were always below 0.25 for the CVS group (Supplementary, Fig. S2) and only slightly above (0.26–0.27) in the non-CVS group. We assumed that having a single, compromise hyperparameter was better than two different ones, i.e., 0.3 for correlation magnitude and 0.2 for correlation consistency, for reasons given in the “Introduction.” As mentioned above, in the subsection “Correlations by CVS status,” loosening the threshold to 0.3 had little effect on the results and the conclusions.

Ideally, the choice of the threshold could have also been done algorithmically, by utilizing a third dataset: We could have performed a systematic search over many possible thresholds, comparing the performance on the training and the validation sets to find the best one and, finally, check the correlated variables on a third test set. Unfortunately, the small amount of data at our disposal did not allow for this approach.

Due to high dimensionality of the data, the relatively small number of observations, and the possibility of random noise appearing as a pattern, the results should be interpreted cautiously. Furthermore, detailed investigations with large numbers of the patients are warranted.

## Conclusion

Clusters of mitochondrial DNA DAMPs are differentially expressed in patients suffering from post-aSAH CVS and those without CVS. As these clusters correlate with the cerebral vasospasm, they may be utilized as potential predictors of it. Basic translational studies investigating the pathophysiological mechanisms and pinpointing the involvement of subcellular pathways underlying these clusters associated with CVS are highly warranted. Finally, our results provide further insights to unentangle the complex pathophysiological mechanisms implicated in the development of CVS, a major contributor to DCI.

### Supplementary Information

Below is the link to the electronic supplementary material.

Figure S1 Heatmap of mean correlations over 10,000 randomly sampled training sets. Variable pairs for which the correlation had the opposite sign in the training set and the validation set in over 5% of the simulation runs are not shown. The lowest observed correlation, 0.26 between IL-23 and Cox-1 gives credibility to the value of 0.25 to be used as the lower bound for a correlation to be considered relevant. (PNG 91 kb)

High resolution image (EPS 802 kb)

Figure S2 Heatmap of mean absolute differences in correlations between 10,000 randomly sampled training and validation sets, for patients who developed CVS. The largest difference is 0.25, for IL-6 and IL-10. (PNG 87 kb)

High resolution image (EPS 803 kb)

Figure S3 Heatmap of mean absolute differences in correlations between 10,000 randomly sampled training and validation sets, for patients who did not develop CVS. All absolute differences are below 0.3. (PNG 82 kb)

High resolution image (EPS 803 kb)

Figure S4 Correlation heatmap of consistently similar and relevant variables for patients who suffered CVS. In contrast to the heatmap shown in Fig. 2, here a loosened criterion for “stable” correlation was used. Instead of 0.25, the absolute differences of correlations between the training and the validation set were allowed to be as high as 0.3, as long as the sign remained the same. Compared to Fig. 2, two additional correlations appear: between age and leukocytes and between age and CCL5. Since age is correlated to IL-6, these two correlations join the already identified cluster consisting of IL-6, IL-10, age, and Hunt-and-Hess score. (PNG 108 kb)

High resolution image (EPS 789 kb)

Figure S5 Correlation heatmap computed over the combined training and validation sets, for patients who developed CVS. As in Fig. S4, leukocytes and CCL5 appears to join the cluster of variables above it. (PNG 112 kb)

High resolution image (EPS 791 kb)

## Data Availability

The datasets analyzed during the current study are available from the corresponding author on reasonable request.
