# Supplementary figures and images for: Clustering of serum biomarkers involved in post-aneurysmal subarachnoid hemorrhage (aSAH) complications
Source: Neurosurg Rev. 2023 Mar 3;46(1):63. doi: 10.1007/s10143-023-01967-9 (PMC9981718; doi:10.1007/s10143-023-01967-9)

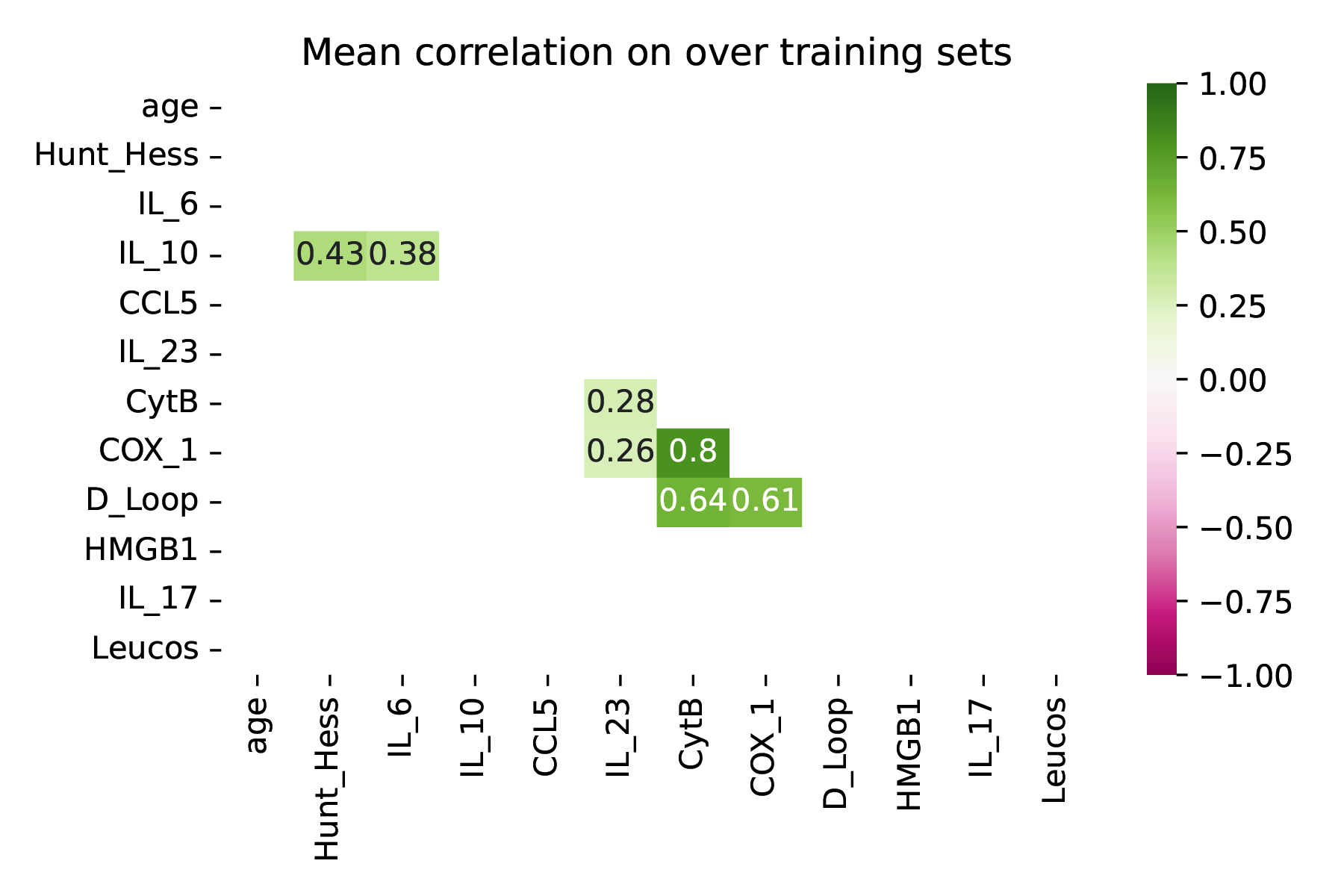

Supplement: Supplementary file 1 — Heatmap of mean correlations over 10,000 randomly sampled training sets. Variable pairs for which the correlation had the opposite sign in the training set and the validation set in over 5% of the simulation runs are not shown. The lowest observed correlation, 0.26 between IL-23 and Cox-1 gives credibility to the value of 0.25 to be used as the lower bound for a correlation to be considered relevant. (PNG 91 kb) [file 10143_2023_1967_Fig6_ESM.png]

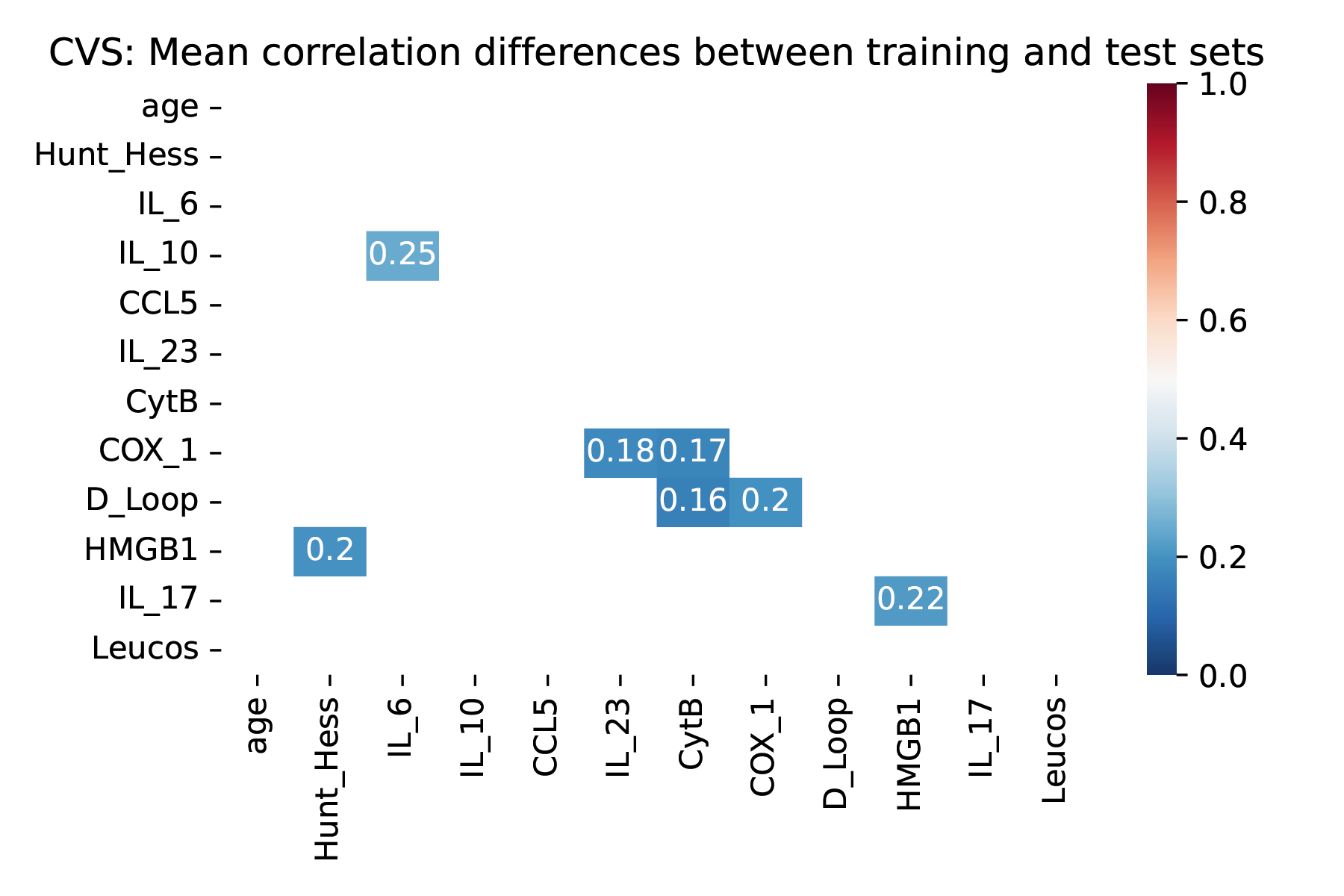

Supplement: Supplementary file 3 — Heatmap of mean absolute differences in correlations between 10,000 randomly sampled training and validation sets, for patients who developed CVS. The largest difference is 0.25, for IL-6 and IL-10. (PNG 87 kb) [file 10143_2023_1967_Fig7_ESM.png]

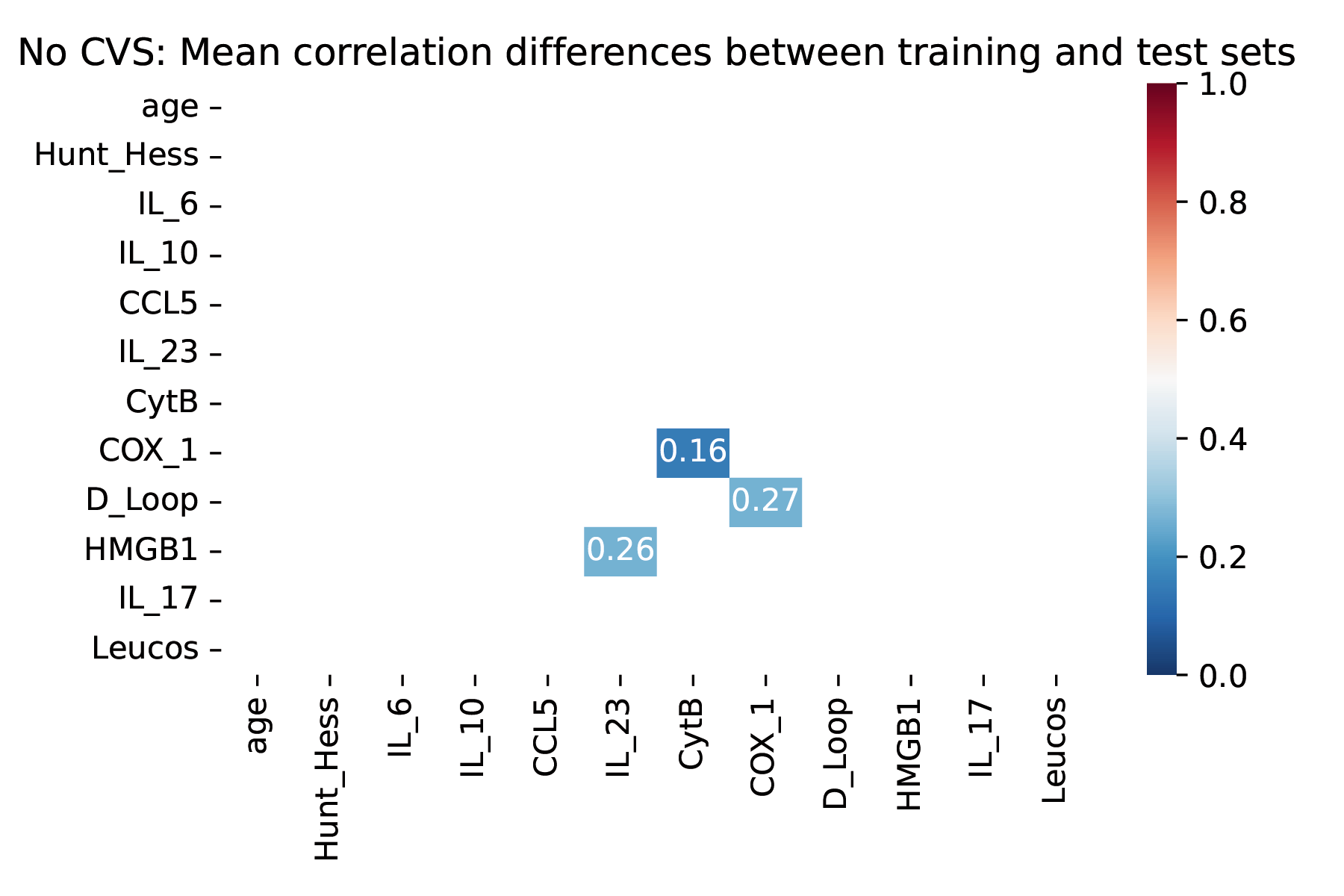

Supplement: Supplementary file 5 — Heatmap of mean absolute differences in correlations between 10,000 randomly sampled training and validation sets, for patients who did not develop CVS. All absolute differences are below 0.3. (PNG 82 kb) [file 10143_2023_1967_Fig8_ESM.png]

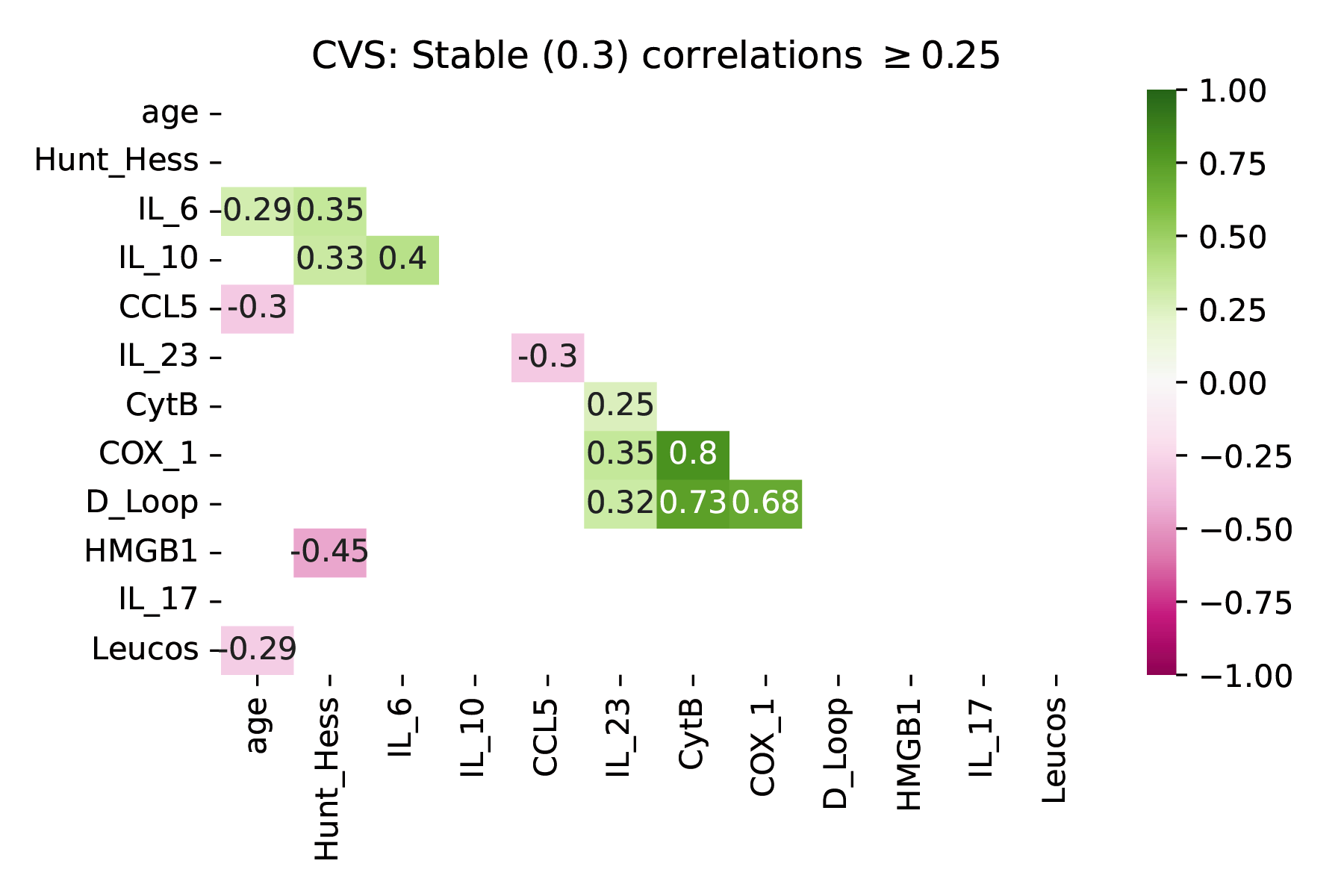

Supplement: Supplementary file 7 — Correlation heatmap of consistently similar and relevant variables for patients who suffered CVS. In contrast to the heatmap shown in Fig. 2, here a loosened criterion for “stable” correlation was used. Instead of 0.25, the absolute differences of correlations between the training and the validation set were allowed to be as high as 0.3, as long as the sign remained the same. Compared to Fig. 2, two additional correlations appear: between age and leukocytes and between age and CCL5. Since age is correlated to IL-6, these two correlations join the already identified cluster consisting of IL-6, IL-10, age, and Hunt-and-Hess score. (PNG 108 kb) [file 10143_2023_1967_Fig9_ESM.png]

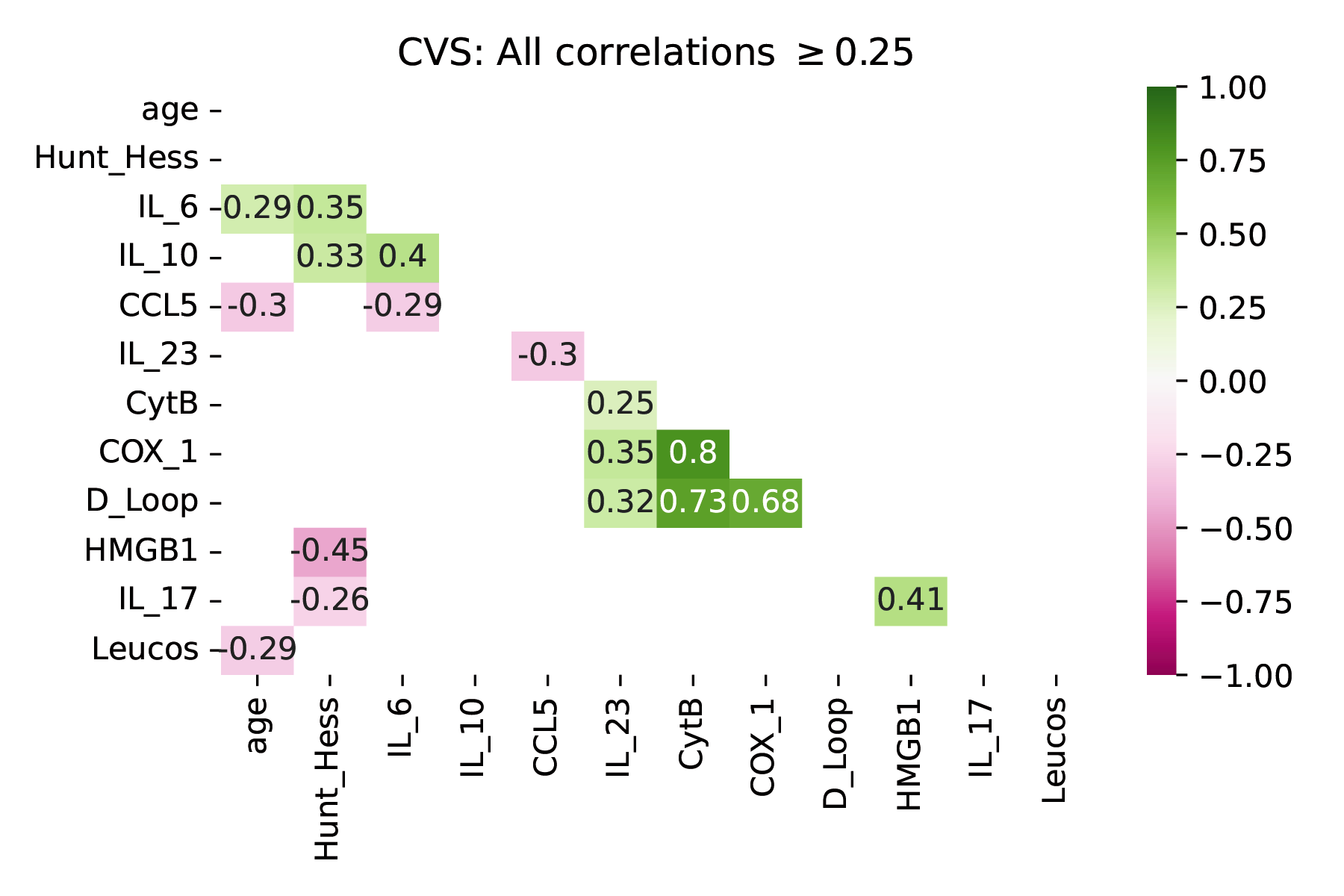

Supplement: Supplementary file 9 — Correlation heatmap computed over the combined training and validation sets, for patients who developed CVS. As in Fig. S4, leukocytes and CCL5 appears to join the cluster of variables above it. (PNG 112 kb) [file 10143_2023_1967_Fig10_ESM.png]
